# DNA and Polyphosphate in Directed Proteolysis for DNA Replication Control

**DOI:** 10.3389/fmicb.2020.585717

**Published:** 2020-10-02

**Authors:** Malgorzata Ropelewska, Marta H. Gross, Igor Konieczny

**Affiliations:** Laboratory of Molecular Biology, Intercollegiate Faculty of Biotechnology of University of Gdańsk and Medical University of Gdańsk, Gdańsk, Poland

**Keywords:** DNA replication, replication initiators, proteolysis, polyphosphate, lon protease

## Abstract

The strict control of bacterial cell proliferation by proteolysis is vital to coordinate cell cycle processes and to adapt to environmental changes. ATP-dependent proteases of the AAA + family are molecular machineries that contribute to cellular proteostasis. Their activity is important to control the level of various proteins, including those that are essential for the regulation of DNA replication. Since the process of proteolysis is irreversible, the protease activity must be tightly regulated and directed toward a specific substrate at the exact time and space in a cell. In our mini review, we discuss the impact of phosphate-containing molecules like DNA and inorganic polyphosphate (PolyP), accumulated during stress, on protease activities. We describe how the directed proteolysis of essential replication proteins contributes to the regulation of DNA replication under normal and stress conditions in bacteria.

## Introduction

Several mechanisms responsible for the control of DNA replication in bacteria were described (Zakrzewska-Czerwińska et al., 2007). Most of those mechanisms aim at decreasing the availability of active replication protein, e.g., by regulating the transcription (Gora et al., 2013), spatial sequestration (Iniesta et al., 2006), or protein inactivation (Kurokawa et al., 1999). It was shown that particular bacterial proteases are involved in the proteolysis of replication proteins and proteins associated with the process of DNA replication (Wickner et al., 1994; Pierechod et al., 2009; Kubik et al., 2012; Karlowicz et al., 2017). The major proteases in bacteria belong to the family of ATPases associated with diverse cellular activities (AAA +). In *Escherichia coli*, there are four cytosolic proteases (i.e., ClpXP, ClpAP, HslUV, and Lon) (Gottesman, 2003). Bacterial AAA + proteases function efficiently under different growth conditions participating in regulation of several cellular processes. For instance, the intracellular levels of the HslUV protease are increased under heat-shock conditions when it has the maximum substrate degradation rate (Burton et al., 2005). In addition to HslUV functions under thermal stress, this protease plays an important role in SOS response caused by DNA damage (Khattar, 1997) and in response to acidic stress (Kannan et al., 2008). ClpXP participates in the response to starvation (Schweder et al., 1996), heat shock, and oxidative stress (Frees et al., 2003). Similarly, ClpAP protease is responsible for the control of regulatory pathways in bacteria and response to proteotoxic stress caused by pH downshift or high temperature (Jenal and Hengge-Aronis, 2003). Lon protease contributes to genome maintenance during stress (e.g., heat shock or nutrient depletion) by regulating DNA replication (Nicoloff et al., 2007; Jonas et al., 2013; Leslie et al., 2015; Gross and Konieczny, 2020). Furthermore, LonA protease is involved in the tolerance of *Actinobacillus pleuropneumoniae* to osmotic or oxidative stress (Xie et al., 2016). Since proteolysis is irreversible, it must be induced at particular conditions and target specific proteins in a tightly controlled manner. Bacterial AAA + proteases are regulated temporally (Goff and Goldberg, 1987; Jonas et al., 2013), spatially (Simmons et al., 2008), and structurally (Jonas et al., 2013) and by interaction with ligand or adaptors (Goldberg et al., 1980; Wah et al., 2002; Martin et al., 2008; Puri, 2016). Proteases interact with various phosphate-containing molecules including membrane components [e.g., lipopolysaccharide (LPS) (Sugiyama et al., 2013) and cardiolipin (CL) (Minami et al., 2011)], stress-induced factors [e.g., guanosine tetraphosphate ((p)ppGpp) (Osbourne et al., 2014) and inorganic polyphosphate (PolyP) (Kuroda, 2006)], ATP (Charette et al., 1981), and ADP (Waxman and Goldberg, 1985) as well as with DNA (Zehnbauer et al., 1981; Zylicz et al., 1998; Kubik et al., 2012). The protease binding to phosphate-containing molecules may change protease localization, ATPase activity, or substrate specificity, thereby modulating its proteolytic activity (Kubik et al., 2012; Karlowicz et al., 2017; Gross and Konieczny, 2020).

## The Impact of DNA Binding on Protease Activity

In *Escherichia coli*, only Lon and ClpAP, but not ClpXP or HslUV, interact with DNA (Kubik et al., 2012). Interaction of Lon with nucleic acid is a conserved property among species (Zehnbauer et al., 1981; Fu and Markovitz, 1998; Lee et al., 2004; Lu et al., 2003). It was demonstrated that the α subdomain in the AAA + module of *Brevibacillus thermoruber* Lon is involved in DNA binding (Lee et al., 2004, 2014; Lin et al., 2009). In various organisms, Lon has different preference for the type of DNA with which it forms a complex. *E. coli* Lon binds to double-stranded DNA (dsDNA) in a sequence-non-specific manner (Charette et al., 1984; Nomura et al., 2004). On the contrary, eukaryotic proteases bind single-stranded DNA (ssDNA) or RNA (Fu and Markovitz, 1998; Lu et al., 2003; Liu et al., 2004). *Bacillus subtilis* LonA is present in the nucleoid under normal growth conditions, while ClpXP is present in cytosol (Simmons et al., 2008). During spore development, LonA changes its localization to the forespore (Simmons et al., 2008). Under heat shock, LonA remains bound to the nucleoid (Simmons et al., 2008). Yet in *E. coli* when temperature is increased, Lon loses its ability to bind DNA *in vitro*, although ATP-dependent proteolytic activity is retained (Sonezaki et al., 1995). It is proposed that the Lon presence within the nucleoid allows for the degradation of DNA-associated proteins involved in DNA metabolism. The protease dissociation from DNA upon stress-related factors may provide rapid adaptive mechanism to hamper Lon activity toward specific proteins (Sonezaki et al., 1995).

The interaction of DNA with Lon stimulates its ATPase activity (Charette et al., 1984). At the surface of *E. coli* Lon ATPase domain, there are located positively charged residues, which are responsible for direct interaction with DNA (Karlowicz et al., 2017). The presence of DNA in a reaction mixture containing Lon and substrate protein enhances protease activity to hydrolyze ATP (Karlowicz et al., 2017). The ATPase activity of Lon mutant defective in DNA interaction is not increased in the presence of substrate and DNA. Hence, it is the direct DNA–Lon interaction that stimulates protease ATPase activity (Karlowicz et al., 2017). It was also demonstrated that Lon nucleoprotein complex formation is essential for the proteolysis of DNA-interacting substrates, but not other substrates (Karlowicz et al., 2017).

The ClpAP proteolysis of DNA-binding substrates is also stimulated by DNA. For example, ParD protein, the component of toxin–antitoxin system of RK2 plasmid (Kubik et al., 2012; Dubiel et al., 2018) is degraded by ClpAP in a DNA-dependent manner (Dubiel et al., 2018). *In vitro* experiments suggest that it is the protease—DNA interaction, but not substrate–DNA interaction, that contributes to the enhanced proteolysis. Although *E. coli* ClpXP and HslUV do not form nucleoprotein complexes, the addition of DNA to the *in vitro* reaction mixture affects the proteolysis of particular substrates (Kubik et al., 2012). As opposed to Lon and ClpAP, the process of proteolysis is inhibited by DNA. This may be explained by the ability of substrates to interact directly with DNA, thus hampering their proteolysis.

## The Impact of Polyphosphate Binding on Protease Activity

When bacteria encounter stress such as amino acid starvation or oxidative stress, they accumulate inorganic PolyP, which forms granular superstructures and contributes to cell survival (Kuroda et al., 2001). The production of PolyP was initially correlated with the synthesis of second messenger stress molecule, (p)ppGpp, which was shown to inhibit the activity of exopolyphosphatase (PPX), thereby enabling uncontrolled production of PolyP by PolyP kinase (PPK) (Kuroda et al., 1997; Magnusson et al., 2005; Traxler et al., 2008; Rao et al., 2009). *Ppk* mutants fail to survive in stationary phase and are less resistant to heat or oxidants (Crooke et al., 1994; Rao and Kornberg, 1996). Recent data argue that (p)ppGpp is not required for PolyP synthesis and that transcription factor DksA contributes to the control of PolyP level instead (Gray, 2019). In *Caulobacter crescentus*, PolyP has been shown to be involved in the regulation of DNA replication during carbon starvation (Boutte et al., 2012). During nitrogen starvation in *Pseudomonas aeruginosa*, PolyP granule biogenesis is temporally and functionally tied to cell cycle exit indicated by the inhibition of reinitiation of DNA replication, completion of open rounds of DNA replication, segregation of daughter chromosomes, and septation (Racki et al., 2017). PolyP interacts with *Escherichia coli* Lon via ATPase domain (Nomura et al., 2004), as in the case of DNA (Karlowicz et al., 2017), which implies that both phosphate-containing molecules can compete for Lon binding. Indeed, the equimolar concentration of PolyP was shown to disrupt the Lon–DNA complex (Nomura et al., 2004) and Lon colocalization with nucleoid (Zhao et al., 2008). Lon loses DNA-binding ability when cells are exposed to heat-shock conditions, which is directly linked to an increase in the amount of damaged proteins (Sonezaki et al., 1995). During starvation, Lon is associated with PolyP granules (Kuroda, 2006). PolyP stimulates Lon to proteolyze ribosomal proteins such as L1, L3, and L24 but inhibits proteolysis of SulA protein (an inhibitor of cell division accumulated in response to DNA damage) (Nomura et al., 2004). When Lon is pre-incubated with PolyP, the proteolysis of L24 ribosomal protein is the most efficient (Nomura et al., 2004). Not all PolyP-interacting proteins are degraded by Lon, but all proteins degraded by Lon in a PolyP-dependent manner do form a complex with PolyP (Kuroda, 2006).

Although a complex of protease with PolyP and its general role was uncovered almost two decades ago, we still lack the full mechanistic and physiological insight into this complex formation. To date, no data are available on how/if PolyP affects other proteases in bacterial cells.

## The Proteolysis of Replication Proteins and Proteins Associated With DNA Replication

Not only proteases but also their substrate can interact with DNA or PolyP. Depending on the substrate, the process of proteolysis is specifically controlled and fine-tuned (Table 1). Here, we discuss the proteolysis of selected replication factors and how it affects cell survival.

**TABLE 1:** Comparison of degradation conditions of replication proteins and proteins associated with DNA replication.

**Substrate**	**Function of a substrate**	**Organism**	**Protease**	**Factors affecting the proteolysis**	**References**
DnaA	Required for bacterial DNA replication initiation	*Caulobacter crescentus*	Lon, ClpAP	Unfolded substrates (+)	Jonas et al., 2013; Liu et al., 2016
		*Escherichia coli*	Lon	PolyP (+)^a^	Gross and Konieczny, 2020
TrfA–wt (dimer)	Participates in the formation of “handcuff” of RK2 plasmid particles	*E. coli*	ClpAP, Lon	DNA (+)^b^	Kubik et al., 2012
			ClpXP, HslUV	DNA (−)	Kubik et al., 2012
TrfA G254D/S256L (monomer)	Participates in replication initiation of RK2 plasmid	*E. coli*	Lon, ClpAP	DNA (+)	Kubik et al., 2012
RepE	Participates in replication initiation of F plasmid	*E. coli*	Lon	DNA (+)	Karlowicz et al., 2017
λO	Participates in replication initiation of phage lambda	*E. coli*	Lon	DNA (+)	Karlowicz et al., 2017
HimA	As a heterodimer with HimD bends DNA in the structure of oriC, thus facilitating the replication initiation	*E. coli*	Lon	PolyP (+)	Nomura et al., 2004
Dps	Protects DNA during starvation and oxidative stress	*E. coli*	ClpAP, ClpXP	?^c^	Stephani et al., 2003
CspD	Inhibits DNA replication; plays a regulatory role in chromosomal replication in nutrient-depleted cells	*E. coli*	Lon	?	Langklotz and Narberhaus, 2011
CtrA	Controls transcription and inhibits DNA replication initiation.	*C. crescentus*	ClpXP	?	Jenal and Fuchs, 1998
CcrM	Inhibits DNA replication initiation	*C. crescentus*	Lon	DNA (+)	Gonzalez et al., 2014
DnaX	Participates in the loading of sliding clamp	*C. crescentus*	ClpXP	?	Vass and Chien, 2013
SocB	Binds to sliding clamp and inhibits elongation of DNA replication	*C. crescentus*	ClpXP	?	Aakre et al., 2013

*^*a*^(+) indicates that the proteolysis is stimulated by DNA, PolyP, or unfolded substrates. ^*b*^(−) indicates that the proteolysis is inhibited by DNA, PolyP, or unfolded substrates. ^*c*^? indicates that there are no data about impact of DNA, PolyP, or unfolded substrates.*

### Replication Initiators

**FIGURE 1:**
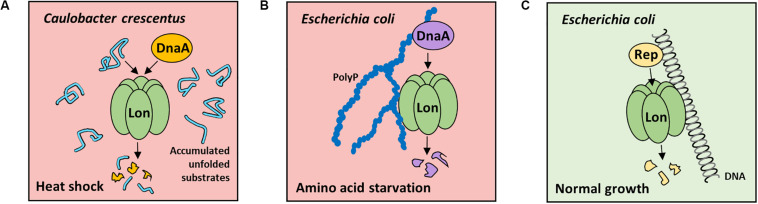
Factors stimulating Lon-dependent proteolysis of replication initiators in *Caulobacter crescentus* and *Escherichia coli* during stress or normal growth conditions. **(A)** In *C. crescentus* in heat-shock conditions, unfolded substrate accumulation stimulate Lon protease for DnaA degradation, which results in inhibition of DNA replication initiation. **(B)** In *E. coli* cells during amino acid starvation, PolyP-induced DnaA proteolysis (PDAP) is launched. PolyP activates Lon protease to degrade DnaA, thereby resulting in the decreasing DnaA level and, consequently, DNA replication initiation arrest. **(C)** Under normal growth conditions, plasmid replication initiation protein (Rep) degradation by Lon is induced by nucleoprotein complex formation. The protease and substrate interaction with DNA is crucial for efficient degradation. No data are available about Rep proteins stability in stress conditions.

The replication initiation proteins are the prerequisite factors responsible for initiating DNA replication in various replicons; thus, their degradation allows for rapid arrest of DNA replication. The DnaA, a highly conserved replication initiation protein in bacteria, is an obvious target for cellular proteases. In *Caulobacter crescentus*, DnaA protein is degraded mainly by Lon, under optimal and stress conditions (Gorbatyuk and Marczynski, 2005; Jonas et al., 2013; Leslie et al., 2015; Liu et al., 2016). It was demonstrated that the DnaA intracellular levels depend on a reduction in DnaA synthesis and fast degradation by the Lon protease. Constitutively, ATP-bound DnaA mutant was shown to be degraded more slowly than wild-type (wt) protein, indicating that degradation of DnaA is linked to DnaA activity or DnaA nucleotide bound state (Liu et al., 2016). Under proteotoxic stress, DnaA is degraded as a result of allosteric activation of Lon by accumulated unfolded substrates and increase in Lon intracellular concentration (Jonas et al., 2013; Figure 1A). Under normal growth conditions, *C. crescentus* DnaA is proteolyzed at the end of S-phase to ensure that only newly synthesized DnaA is available at the start of each replication round (Jenal, 2009). The overexpression of ClpA in *lon*-depleted strain restores DnaA degradation, indicating that fail-safe systems are present (Liu et al., 2016).

The regulatory mechanism that controls DNA replication in *Escherichia coli* by directed proteolysis of replication initiator was termed PolyP-induced DnaA proteolysis (PDAP) (Gross and Konieczny, 2020; Figure 1B). In *E. coli* cells during amino acid starvation, PolyP induces Lon activity to specifically degrade, DnaA when bound to ADP, but not ATP. When PolyP-synthesizing enzyme (PPK) or Lon protease is depleted in *E. coli* during stress, DnaA level remains high. Also, the level of DnaA protein variant permanently bound to ATP does not change in stress conditions (Gross and Konieczny, 2020). Both *in vivo* and *in vitro* data indicate that when DnaA is converted to ADP-bound form, it is degraded by Lon (Gross and Konieczny, 2020). PolyP interacts with DnaA-ADP, but not DnaA-ATP, which provides an explanation on how Lon targets only DnaA-ADP for proteolysis. In starvation, as a result of an increase in Lon level and Lon activation by PolyP, the overall DnaA concentration decreases, which leads to the inhibition of DNA replication initiation (Gross and Konieczny, 2020). Since in *E. coli* (Gross and Konieczny, 2020) and in *C. crescentus* (Liu et al., 2016) DnaA protein degradation depends on its nucleotide state, it may be crucial for the control of DNA replication. Such possibility is discussed in a recent review on the regulation of *Caulobacter* DnaA (Felletti et al., 2019). It was also shown that in stress in *E. coli*, ppGpp affects RNA polymerase activity and thereby superhelicity of replication origin, which leads to DNA replication initiation inhibition (Kraemer et al., 2019). Because ppGpp is not required for PolyP synthesis in *E. coli* (Crooke et al., 1994), it is very likely that the regulations by ppGpp (Kraemer et al., 2019) and PDAP (Gross and Konieczny, 2020) are independent mechanisms responsible for controlling DNA replication initiation during stress in *E. coli*.

DnaA participates in the replication initiation of many plasmids, which implies that the replication of plasmid and chromosome in one cell may be coordinately regulated by the inducible degradation of DnaA during stress conditions. This possibility requires to be investigated. It was shown that stability of plasmid DNA is decreased in *E. coli* protease-deficient mutants (Bury et al., 2017; Dubiel et al., 2018). Plasmid-encoded replication initiators (Rep), e.g., RK2 plasmid TrfA protein, are degraded by Lon and other cytosolic proteases (Wojtkowiak et al., 1993; Wickner et al., 1994; Levchenko et al., 1995; Pierechod et al., 2009; Kubik et al., 2012). The selective proteases activity may affect Rep monomer/dimer ratio and therefore the ability of replication initiator to initiate plasmid DNA replication. DNA stimulates TrfA degradation by Lon (Figure 1C) and ClpAP but inhibits proteolysis by ClpXP and HslUV (Kubik et al., 2012). Similarly, binding of λO protein, i.e., replication initiator of bacteriophage Lambda, to *ori*λ DNA protects it from degradation by ClpXP (Zylicz et al., 1998). Despite replication initiation control by the Rep concentration and monomer/dimer ratio, the RK2 plasmid replication is also controlled by joining two DNA plasmid particles via TrfA to form handcuff complex, thereby preventing replication reinitiation. *E. coli* Lon disrupts the handcuff complex by proteolyzing TrfA (Bury et al., 2017).

### CtrA

The response regulator CtrA in *C. crescentus* is another DNA-binding protein whose level is controlled by proteases. CtrA not only controls transcription of more than a hundred genes (Wojtkowiak et al., 1993) but also inhibits DNA replication initiation (Quon et al., 1996, 1998; Laub et al., 2002). For replication to occur, CtrA must be eliminated at the G1–S transition, and this is carried out by dephosphorylation (Jacobs et al., 2003) and ClpXP-mediated proteolysis (Jenal and Fuchs, 1998). Under nutritional stress, CtrA proteolysis is inhibited by ppGpp and PolyP accumulation (Boutte et al., 2012). The proteolysis of CtrA is carried out by ClpXP only when both proteins are localized in the cell pole (Iniesta et al., 2006). This process occurs in the presence of accessory proteins, i.e., CpdR, RcdA, PopA, and cyclic diguanylate (cdG), which accelerate CtrA degradation *in vitro*. Those accessory proteins are also essential for proteolysis of CtrA bound to DNA (Smith et al., 2014).

### CcrM

In order to complete cell division, the chromosome needs to be fully methylated by the CcrM DNA methyltransferase. This methyltransferase CcrM is proteolyzed by Lon to restrict CcrM to most of the cell cycle that prolongs the hemimethylation state of chromosomal DNA during DNA synthesis in *C. crescentus* (Wright et al., 1996). The *ccrM* gene transcription is regulated by a positive global regulator CtrA, and the CcrM protein is constitutively degraded by Lon (Wright et al., 1996). Not only DNA was shown to stimulate Lon-mediated proteolysis of CcrM but also CcrM has 10-fold higher affinity for Lon in the presence of DNA, when compared with CcrM to Lon alone (Zhou et al., 2019). The C-terminus of CcrM binds DNA and is recognized by Lon (Zhou et al., 2019). Lon interaction with DNA is not crucial for CcrM proteolysis because CcrM degradation is still observed in cells expressing Lon mutant defective in DNA binding (Zeinert et al., 2018). Therefore, the CcrM level and correct completion of cell cycle depend on the balance between the synthesis and proteolysis of CcrM. CcrM degradation by Lon can also affect the dNTP production in a cell. In Δ*lon* strains, an increase in the ribonucleotide reductase (RNR) expression level is observed, which is driven by stabilization of the transcription factor CcrM (Zeinert et al., 2020).

### Integration Host Factor

The integration host factor (IHF) (Nomura et al., 2004) is a histone-like protein responsible for modulation of the DNA condensation (Pettijohn, 1988). IHF is a HimA/HimD heterodimer, which interacts with DNA through specific binding sequence (IBS, IHF binding sequence) and bends DNA in the structure of *oriC*, thus facilitating the process of replication initiation in *E. coli* (Ozaki and Katayama, 2012). IHF also participates in regulating the nucleotide state of DnaA. IHF dimers bound to *datA* sequence promote DnaA-ATP hydrolysis in the DDAH system, thus increasing the pool of DnaA-ADP to prevent overinitiation (Kasho and Katayama, 2013). Moreover, IHF, together with Fis, binds to DARS2 sequence and participates in DnaA-ATP regeneration, which is coupled to cell cycle and growth phase (Kasho et al., 2014). IHF interacts with PolyP (Kornberg, 1995), and its level is regulated by Lon in a PolyP-dependent manner (Nomura et al., 2004). The IHF oligomeric state has an impact on this process. HimA degradation is dependent on PolyP and Lon, as opposed to HimD. When both monomers formed heterodimers, neither HimA nor HimD is degraded (Nomura et al., 2004). This suggests that either Lon recognition for HimA is buried at the interface of monomers within heterodimer or a significant structural rearrangement occurs upon dimerization.

### CspD

Upon entry into the stationary phase in *E. coli*, CspD is expressed and acts as an inhibitor of replication (Yamanaka and Inouye, 1997). Expression of CspD was shown to be activated by (p)ppGpp (Yamanaka and Inouye, 1997). This allows for the adaptation to nutritional changes. CspD was found to be related to persister cell formation (Kim and Wood, 2010). Cellular level of CspD is regulated in response to growth phase and growth rate by proteolysis. Using electron microscopy (EM), it was shown that CspD condenses ssDNA; however, those nucleoprotein complexes are distinct from the complex of single-stranded binding protein (SSB) with DNA (Yamanaka et al., 2001). When growth is resumed in nutrient-rich environment, CspD is degraded by Lon (Langklotz and Narberhaus, 2011). The proteolysis of CspD by Lon was reconstituted *in vitro* and did not require any additives, besides ATP, which indicates that during growth, unknown factors must regulate either Lon activity or CspD availability for degradation.

### Dps

Known as the most abundant protein in a stationary phase in *E. coli*, Dps was shown to protect DNA during starvation and oxidative stress, by self-aggregation and DNA condensation (Almirón et al., 1992; Azam et al., 1999; Ceci et al., 2004; Frenkiel-Krispin et al., 2004; Melekhov et al., 2015). During DNA damage, Dps also interacts with DnaA in order to delay replication initiation and allow for DNA repair (Chodavarapu et al., 2008). ClpAP and ClpXP degrade Dps during the exponential phase, which leads to a significant reduction in Dps level (Ninnis et al., 2009). Considering the involvement of Dps in various important functions, its level must be tightly controlled.

### SocB

Sliding clamp (a protein responsible for the replisome processivity in DNA replication) is inhibited by SocB, a component of SocB toxin–SocA antitoxin system in *C. crescentus* (Aakre et al., 2013). The SocB is unstable and constitutively proteolyzed by ClpXP in the presence of SocA. SocB interacts with sliding clamp and inhibits elongation of DNA replication, presumably by outcompeting other proteins from binding sliding clamp. The excessive sliding clamp occupation by SocB leads to premature collapse of replication fork and incomplete cell cycle (Aakre et al., 2013).

### DnaX

For the sliding clamp to be loaded onto DNA, a clamp loader complex is required. In *E. coli*, this complex contains tau and gamma subunits, which are produced from the same gene, but gamma is shorter due to ribosomal frameshifting (Lee and Walker, 1987). In *C. crescentus*, which lacks a frameshifting site, ClpXP generates the shorter version, i.e., gamma subunit, which is necessary under normal growth conditions as well as for DNA damage tolerance (Vass and Chien, 2013).

## Conclusion and Perspectives

In this review, we highlight that directed proteolysis can be stimulated by protease interaction with phosphate-containing molecules such as DNA and PolyP. To date, no structural data are available on such complexes. This specific interaction affects protease activity and selectivity against substrates especially those important in regulation of DNA replication. The current knowledge indicates that among all cytosolic proteases, Lon plays the most important role in the regulation of DNA replication in bacterial cells. We propose that during normal growth, it is the nucleoid DNA that provides matrix for Lon and its substrate proteins. During stress, Lon binds to PolyP granules, thereby stimulating degradation of substrates, which also interact with PolyP in stress. The exact molecular mechanism for this activation remains to be elucidated and needs further validation. Application of the cutting-edge structural research, single-molecule experiments, and trapping approach (Aubin-Tam et al., 2011; Arends et al., 2018; Hu et al., 2018; Fei et al., 2020) should provide insight into the structure–function relationship of Lon, its substrates, adaptor proteins, and complexes with phosphate-containing molecules. Growing evidence indicates that proteolysis is crucial for virulence in many pathogens (Butler et al., 2006; Ingmer and Brøndsted, 2009; Willett et al., 2015). Understanding how directed proteolysis is regulated by phosphate-containing molecules will give insight into microbial stress responses and the regulation of DNA replication.

## Author Contributions

MR, MG, and IK compiled the concept of the mini review. MR and MG wrote the initial version of text. MR and IK prepared the figure and table and wrote the final version of the manuscript. All authors contributed to the article and approved the submitted version.

## Conflict of Interest

The authors declare that the research was conducted in the absence of any commercial or financial relationships that could be construed as a potential conflict of interest.
